# Numerical Design Calculation According to EN 1993-1-14 of Innovative Thin-Walled Columns with Sectional Transverse Strengthening

**DOI:** 10.3390/ma18163878

**Published:** 2025-08-19

**Authors:** Szymon Szewczyk, Volodymyr Semko, Robert Studziński

**Affiliations:** Faculty of Civil and Transport Engineering, Poznan University of Technology, Piotrowo 5 Street, 60-965 Poznan, Poland; szymon.szewczyk@student.put.poznan.pl (S.S.); volodymyr.semko@put.poznan.pl (V.S.)

**Keywords:** cold-formed thin-walled profiles, numerical modelling, Eurocode 3, geometrical imperfections, geometrically nonlinear analysis

## Abstract

This paper presents a numerical analysis of cold-formed thin-walled columns reinforced with sectional transverse stiffeners (STSs) based on the recent part of EC3 concerning the finite element analysis. Columns that are 1 m tall with various arrangements of STSs were modeled in the AxisVM environment. Numerical design calculations were completed using an analysis requiring a subsequent design check. This included a geometrically nonlinear analysis considering imperfections (GNIA) along with linear analysis (LBA) to assess the columns’ susceptibility to second-order effects. Reinforcing columns with STSs did not show a significant effect on the local buckling behavior of the elements. However, the results indicated that increasing the number of STSs positively influenced the columns’ resistance. This modification reduced the magnitudes of distortional, global flexural, and torsional buckling. Additionally, adding more than three STSs increased the critical loads related to distortional, flexural, and torsional buckling by 58–90%, 52–119%, and 19–154%, respectively. For the GNIA, two combinations of imperfections were analyzed: global flexural imperfection paired with either local or distortional imperfection. LBA was used to apply the imperfect geometry of the columns with the appropriate magnitudes of imperfections. The results between LBA and GNIA for the single-branched columns varied by 8–24%, while for the double-branched columns, the differences were less than 3%.

## 1. Introduction

The Eurocodes constitute a harmonized set of European standards for the structural design of buildings and civil engineering works. Developed by the European Committee for Standardization (CEN), these standards are intended to ensure the safety, serviceability, and durability of structures while also supporting the internal market by providing a common technical language for designers, contractors, and regulatory authorities across Europe. Since their initial publication in the early 2000s, the Eurocodes have become a foundational element in structural design practice within the European Union and beyond.

Over the past two decades, there have been substantial advancements in structural analysis techniques, materials science, and computational capabilities. In response to these developments, the second generation of Eurocodes has been introduced. This revision process aims to improve the clarity, consistency, and technical content of the original documents, and to reflect modern design practices, with particular emphasis on performance-based approaches and digital methods. Among the most significant additions to the second generation is the introduction of guidelines for design assisted by finite element analysis (FEA).

The new standard EN 1993-1-14 [1], part of Eurocode 3, provides guidance on the application of numerical methods in the design of steel structures, specifically addressing verifications of the ultimate limit state, fatigue design situations, and the serviceability limit state. It also outlines the use of advanced finite element and related modeling techniques for numerical simulations, including those used in safety assessments. The new standard EN 1993-1-14 [1] addresses design assisted by FEA and reflects the anticipated broader adoption of finite element methods (FEM) in future steel structure design [2]. The standard outlines general methodologies for the modeling, analysis, and design of steel structures, including approaches such as the finite element method (FEM), finite strip method (FSM), and generalized beam theory (GBT). The standard applies to a wide range of structural components and joint configurations, such as hot-rolled, cold-formed, welded plated, and stainless steel profiles, as well as plate assemblies, shell structures, and both welded and bolted joints and connections. It compiles guidelines formerly found in other parts of the Eurocode (e.g., Annex C of EN 1993-1-5 [3]).

The standard provides a unified approach to FEA. It allows taking geometric and material properties as nominal. Terms such as validation and verification are defined. Verification is a comparison of the numerical solutions and accurate analytical or numerical results. Validation is a comparison of the numerical solution and the experimental behavior (or known accurate solutions).

Generally, EN 1993-1-14 [1] distinguishes two methods of FEA: numerical design calculation (NDC) and numerical simulation (NS). NDC involves the use of numerical models and analysis techniques to perform static design checks on a structure or its components. The outcomes of such analyses may include system response quantities (SRQs) for subsequent evaluation or direct resistance values that can be applied directly in static verification. NS serves as a complement or alternative to physical testing for determining the direct resistance of structural components.

Cold-formed thin-walled (TWCF) steel elements are produced by folding thin sheets of steel at ambient temperature. This technology allows for the creation of a variety of cross-sections that can serve different purposes according to the market demand. TWCF elements are used in civil engineering as purlins, columns, rafters, framing, racks, sheeting, and decking, along with numerous other applications [4,5,6,7]. The low weight together with the versatility of TWCF elements results in an up-and-coming alternative to other structural materials, especially with the current demand for sustainable solutions and development of modular construction [8,9]. However, their susceptibility to buckling, particularly under compressive loads, remains a critical design challenge. The basis for design calculations for cold-formed elements is found in EN 1993-1-3 [10]. A significant portion of calculations is based on the effective width concept broadly described in EN 1993-1-5 [3].

The scientific and engineering environment broadly researches ways of coping with the susceptibility of TWCF elements to compression loads. The study by [11] presents an optimization of built-up column cross-sections using machine learning. The aim was to improve the load-bearing capacity without changing the material used. Finite element analysis indicated that the strength of the optimized closed and open cross-sections increased by 21% and 91%, respectively. In [12], reinforcement of TWCF columns with plain channel sleeves connected by screws to the column is presented. The authors prepared numerical models with different configurations of sleeve spacing, column length, and eccentricity of the compressive load. Results of this research demonstrated the effectiveness of the solution in restraining or inhibiting the occurrence of post-distortional buckling. In a study by [7], the authors propose applying inserting sectional transverse stiffeners into double-lipped flanges of the cross-section and joining them with an innovative point crimping method not used in civil engineering applications previously. The findings indicate a notable enhancement in the beams’ performance when subjected to concentrated loads. An efficient way to enhance TWCF elements’ stability and bearing capacity is to create a built-up section by joining multiple single profiles with fasteners, welds, or both [13]. Despite their promising performance, the design rules for built-up sections are not covered by the standards. For this reason, built-up sections are investigated [14,15,16,17,18,19,20,21,22].

Numerical modeling plays a key role in assessing the structural performance of these optimized sections, enabling them to predict their behavior under different loading conditions accurately. By integrating numerical simulations with standardized design procedures, this research contributes to the advancement of thin-walled column design, offering insights into improved buckling resistance and load-carrying capacity. The findings aim to support engineers in adopting optimized structural solutions while adhering to established Eurocode provisions.

This paper presents numerical design calculations of thin-walled columns reinforced with sectional transverse strengtheners joined by the point crimping method. The main goal of the study is to utilize procedures stated in EN 1993-1-14 [1] regarding numerical design calculations in order to predict the behavior of the TWCF columns reinforced with STS elements. The results of the study will provide a basic background for future experimental studies regarding the double-branched thin-walled columns with sectional transverse strengthening.

## 2. Materials and Methods

**Figure 1:**
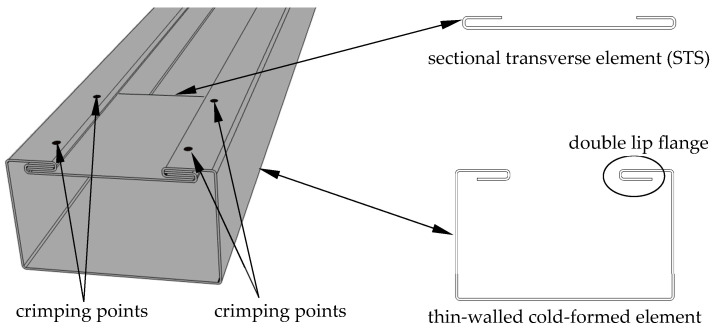
Schematic representation of the point crimping method.

The concept of reinforcing TWCF elements with sectional transverse strengtheners (STSs) connected by the point crimping method (PCM) presented was introduced through a collaboration between researchers at Poznan University of Technology and ARPSTAL Antoni Ratajczak company (Poznań, Poland). The visualization of the method is shown in Figure 1.

The PCM method involves the creation of a cross-section with a double lip on the flanges, which allows the STS element to be inserted into the gap created by the lip. The STS element is a metal sheet of the same material as the main element (e.g., beam column), with two bends at its ends that enable the possibility to interlock with the double lips of the TWCF element flanges. Such a structural solution enhances the load-bearing capacity by locally closing it, which improves its torsional strength as well as counteracts the distortional buckling. The innovative aspect of this solution is the elimination of welds and bolts in the connection system. To the authors’ knowledge, crimping has not yet been used to join structural elements in construction applications. This kind of connection method can be executed with a custom device for sheet clamping developed by the authors [7]. Such a tool forms an indentation in the double-lip flange of the cross-section and crimps it together with the double lip of the STS.

**Figure 2:**
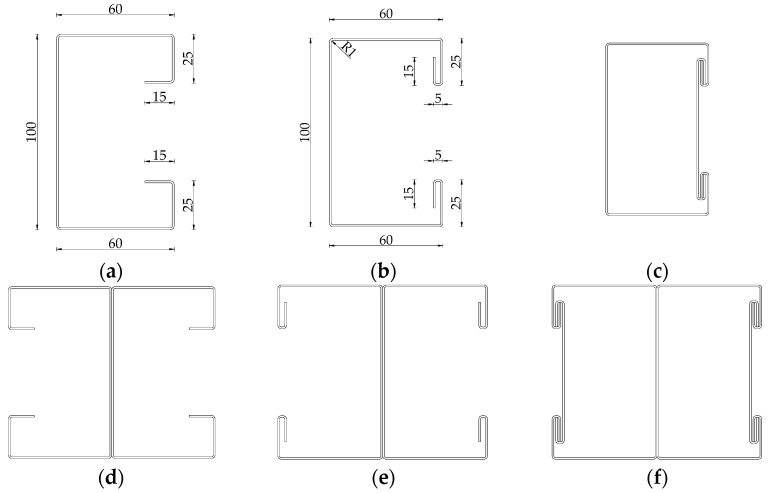
Sample cross-sections: (**a**) single with perpendicular double lip, dimensions in millimeters; (**b**) single with parallel double lip without STSs, dimensions in millimeters; (**c**) single with parallel double lip with STSs applied; (**d**) single with perpendicular double lip; (**e**) single with parallel double lip without STSs; (**f**) single with parallel double lip with STSs applied.

Figure 1 describes the concept of the PCM method of joining a single TWCF element. This study also takes into consideration open double-branched (built-up) elements consisting of two single-channel profiles connected back-to-back, as shown in Figure 2d–f. Figure 2a,b display the basic dimensions of cross-sections in millimeters. The overall height of the cross-section is 100 mm, the width is 60 mm long, and the dimensions of the edge folds are 25 and 15 mm. The internal radius of the bend is 1 mm.

### 2.1. Numerical Model

For the study, a total of 8 samples, see Table 1, 4 single-branched (A–D) as well as 4 double-branched (A2–D2) columns with different numbers of STSs, were modeled in the structural analysis software AxisVM (Version, X8) [23]. Each column is 100 cm tall. Each STS element is 10 cm long.

The following FEA methodology was adopted in this study. The finite element model was developed in the AxisVM environment. For each type of test sample, the geometry, materials, loads, and boundary conditions were modeled. Stability of the meshed samples was checked using LBA analysis. Geometric imperfections were included by using the buckling modes resulting from the LBA analysis, scaling them accordingly to requirements stated in EN 1993-1-14 [1] and EN 1993-1-1 [24], and applying them to the geometry. Nonlinear static analysis was performed, and von Mises stresses were read and compared. To sum up, the finite element simulations were solved using the geometrically nonlinear analysis considering imperfections (GNIA) in conjunction with linear buckling analysis (LBA).

**Figure 3:**
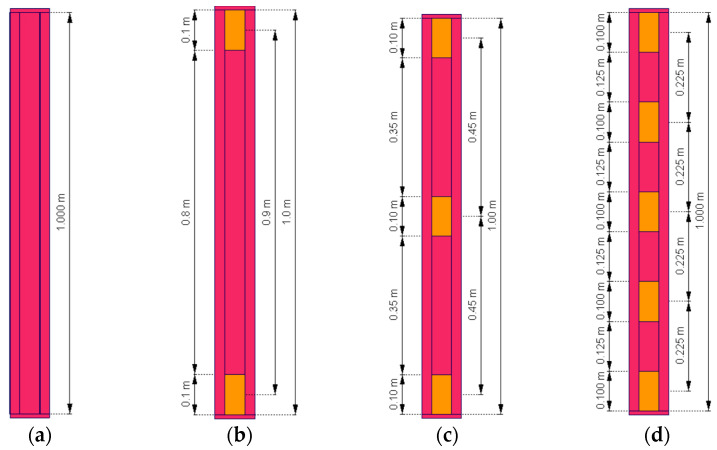
Arrangement of the STSs in tested thin-walled columns: (**a**) No STS; (**b**) 2 × 0.1 m; (**c**) 3 × 0.1 m; (**d**) 5 × 0.1 m.

Figure 3a–d present the arrangements of STSs used. All elements—columns, STSs, and end plates—are assumed to be made of S320GD steel. The thickness of the cold-formed columns and STS elements is 1 mm. End plates at the top and bottom of the column are 10 mm thick. For this study, a shell model of each column, listed in Table 1, was created. Boundary conditions are presented in Figure 3. Each column was loaded with a vertical nodal force of value 25 kN, located in the geometrical center of the top plate. The given position of the load corresponds to the experimental conditions of the future test bed.

**Figure 4:**
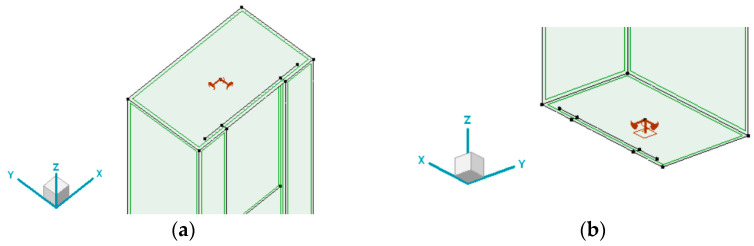
Boundary conditions: (**a**) top plate, nodal u_X_ = u_Y_ = θ_Z_ = 0; (**b**) bottom plate, nodal u_X_ = u_Y_ = u_Z_ = θ_Z_ = 0.

In the numerical model, the following boundary conditions were defined, as shown in Figure 4. Nodal support at the top plate restrains horizontal displacements as well as the torsional rotation of the column. Nodal support at the base of the column additionally restrains vertical displacement. Such boundary conditions aim to resemble a future test bed.

For both static and buckling analyses, the elements were modeled using shell finite elements available in AxisVM software. This element type is based on Mindlin–Reissner theory, meaning that transverse shear deformation effects are allowed. The variation in internal forces within an element can be regarded as linear [23]. Elements were discretized by a finite element mesh consisting primarily of quadrilateral eight/nine-node finite elements.

**Figure 5:**
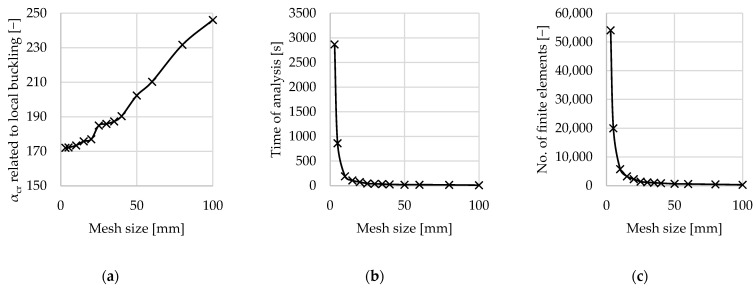
Plots illustrating the quantitative influence on (**a**) the critical load multiplier for the first buckling mode (with 25 modes calculated); (**b**) the analysis time; (**c**) the number of finite elements.

In the finite element model, a mesh size of 20 mm was adopted for both linear buckling analysis (LBA) and geometrically and materially nonlinear analysis considering imperfections (GMNIA). A convergence study preceded the mesh size selection. The evaluation was carried out on a single-branch column without sectional transverse stiffeners (STSs). The influence of the mesh size on the critical load multiplier for the first buckling mode (with 25 modes requested in the analysis) was assessed, along with the corresponding analysis time, output file size, and total number of finite elements. The quantitative results are summarized in Table 2 and presented graphically in Figure 5. In the plots shown in Figure 5, the horizontal axis represents the mesh element size.

**Figure 6:**
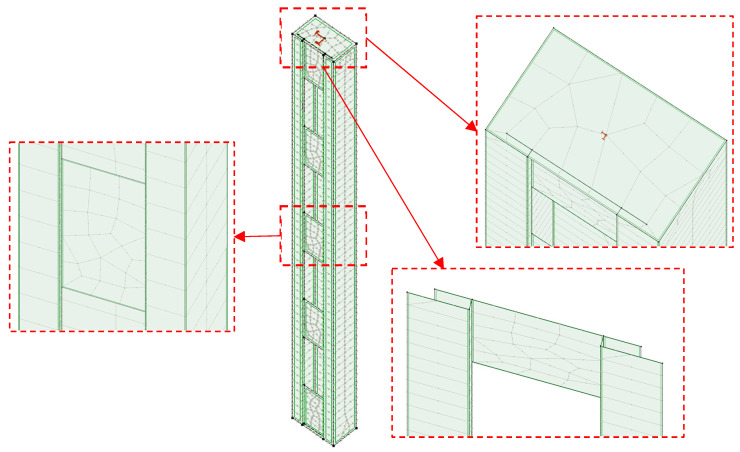
Meshing of the numerical model.

The results indicate an exponential increase in computational cost for mesh sizes smaller than 10 mm. The difference between the 10 mm mesh and the 20 mm mesh adopted in the numerical experiment described in this study—when considering the evaluated parameter (the critical load multiplier for the first buckling mode)—is negligible. Therefore, the 20 mm mesh size (see Figure 6) was deemed appropriate for subsequent analyses.

### 2.2. Imperfections in Finite Element Analysis

As mentioned in EN 1993-1-14 [1], imperfections in the FE model should consider the effects of geometric deviations from perfect shape, residual stresses, and boundary condition defects. The nature of imperfections is random and comes from different stages of the element’s life cycle, such as manufacturing, storage, shipping, and construction [25,26]. Imperfections in the FE model should be implemented either as geometric imperfections and additional residual stresses due to fabrication or equivalent geometric imperfections by modification of the perfect shape of the structure. Among others, one way of defining geometric imperfections in an FE model is through linear buckling analysis (LBA). It means scaling the buckling mode resulting from LBA by an appropriate magnitude and applying it to the model. This method resembles both geometrical imperfections and residual stresses. The standard provides shapes and magnitudes for various kinds of imperfections and elements.

In order to define the range of critical loads associated with different types of stability loss (local, distortional, flexural, and lateral–torsional), linear buckling analysis (LBA) was performed. The benefits of adding STSs to the columns can be determined by comparing critical load factors $\alpha_{cr}$. In the case of the examined columns, most of the buckling modes showed either local buckling or the interaction of local buckling with either distortional, flexural, or lateral–torsional buckling. In order to achieve a “clean” global buckling mode (without local buckling of the walls of either the web or flanges of the column), one should request a relatively greater number of buckling modes with a coarse mesh, which in the case of the examined columns can lead to problems with convergence.

Numerical design calculations are divided into two categories in terms of methods of analysis. Analysis requiring a subsequent design check relates to LA (linear analysis), LBA, GNA (geometrically nonlinear analysis), GNIA (geometrically nonlinear analysis considering imperfections), and MNA (materially nonlinear analysis). This approach generates intermediate results which need to be further used in standard design checks. Direct resistance check relates to MNA, GMNA (geometrically and materially nonlinear analysis), and GMNIA (geometrically and materially nonlinear analysis considering imperfections). The result of this approach is the ultimate resistance of the analyzed structure.

In order to perform an analysis requiring a subsequent design check, a GNIA analysis was conducted. The following two kinds of imperfection combinations were considered in the study:

1. Local imperfections combined with global bow imperfection;
2. Distortional imperfection combined with global bow imperfection.

The influence of second-order effects will be measured by the difference in the reduced von Mises stresses measured in the finite element midplane.

**Figure 7:**
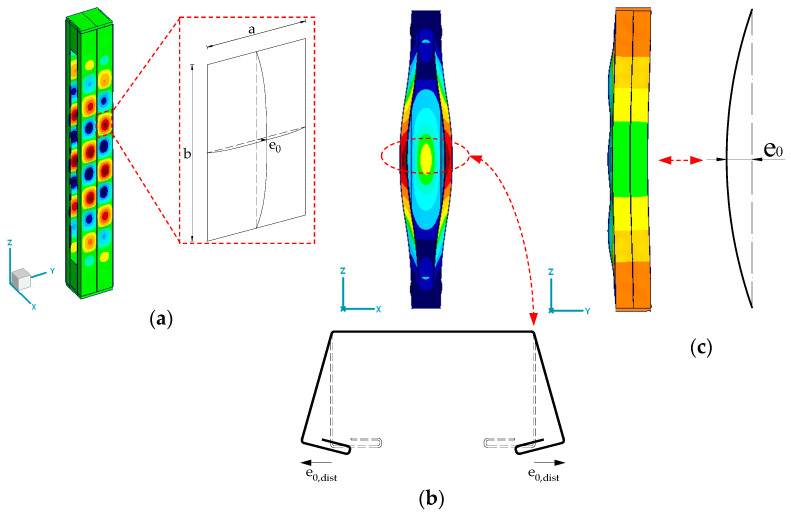
Shapes of geometric imperfections included in the study: (**a**) local buckling of panels and sub-panels; (**b**) distortional buckling; (**c**) global bow imperfection.

According to [1,25], different shapes of local imperfections are defined. For panels and sub-panels (Figure 7a), the magnitude $e_{0}$ is equal to the smaller of a/200 and b/200, where a and b are the dimensions of the panels. The distortional buckling of cold-formed sections (Figure 7b) can be calculated with Formula (1). The global bow imperfection of the member (Figure 7c) was calculated with Formula (2).

Distortional imperfection was calculated according to Formula 5.17 of EN 1993-1-14 [1], as follows:

(1) $$e_{0,dist}=0.3\cdot t\cdot \sqrt{\frac{f_{yb}}{\sigma_{cr,d}}}$$

where $f_{yb}$ is the basic yield strength according to EN 1993-1-3 [10], t is the thickness of the sheet, and $\sigma_{cr,d}$ is the elastic critical distortional buckling stress. The critical stress value used in calculations was the longitudinal stress in the midplane of the finite element resulting from linear analysis, multiplied by the appropriate $\alpha_{cr,dist}$ load factor resulting from linear buckling analysis.

Bow imperfection of the column was determined according to Formula 7.9 of EN 1993-1-1 [24], as follows:

(2) $$e_{0}=\frac{\alpha }{\varepsilon }\beta L$$

where α is the imperfection factor depending on the relevant buckling curve according to Table 8.2 of [25], ε is the material parameter defined in clause 5.2.5 (2) of [24], β is the reference relative bow imperfection according to Table 7.1 of [25], and L is the member length.

The Eurocodes [1,24] define equivalent bow imperfection for lateral torsional buckling for members in bending; however, currently there are no rules for imperfections related to the twist of the element. For that reason, as well as high critical load factors related to the twisting of the columns observed in LBA analysis, the torsional imperfections were omitted in the study.

## 3. Results

Table 3 summarizes the results of the linear buckling analysis of each single-branched (A–D) and double-branched (A2–D2) column. The following kinds of buckling modes were associated with elastic critical load factors ($\alpha_{cr}$): first local buckling mode ($\alpha_{cr,l}$), first distortional buckling mode ($\alpha_{cr,dist}$), first flexural buckling mode ($\alpha_{cr,b}$), and first torsional buckling mode ($\alpha_{cr,t})$.

**Figure 8:**
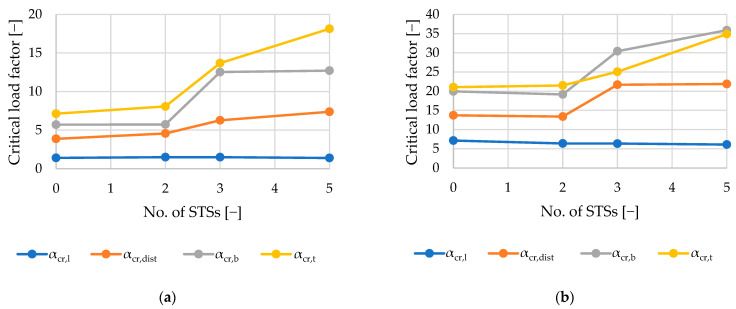
Diagrams of variability of the critical load factors: (**a**) single-branched columns; (**b**) double-branched columns.

**Figure 9:**
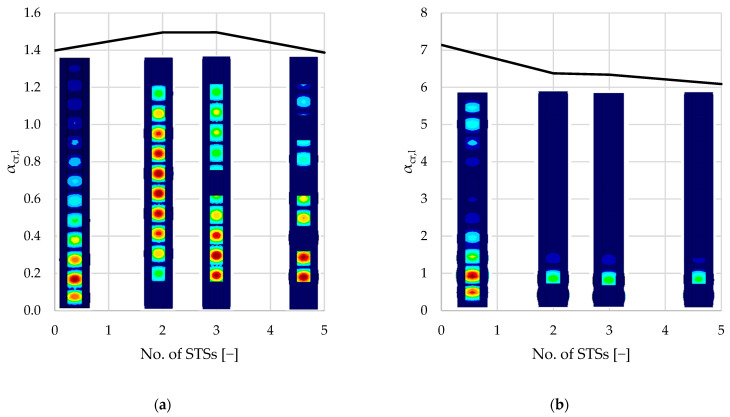
Diagrams and shapes of local buckling modes: (**a**) single-branched columns; (**b**) double-branched columns.

**Figure 10:**
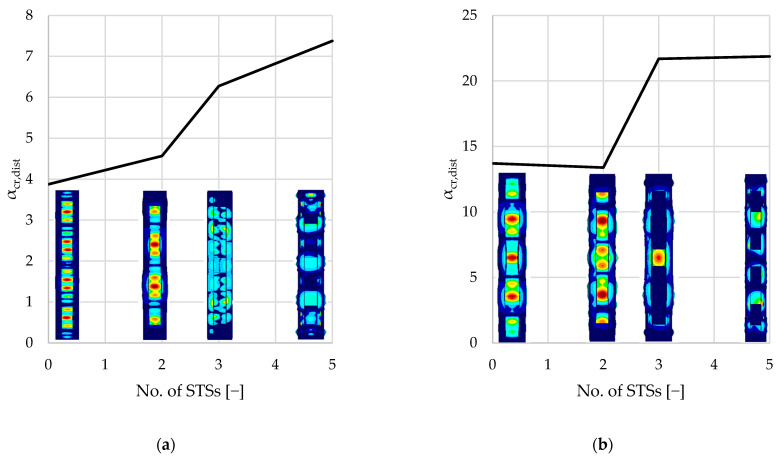
Diagrams and shapes of distortional buckling modes: (**a**) single-branched columns; (**b**) double-branched columns.

**Figure 11:**
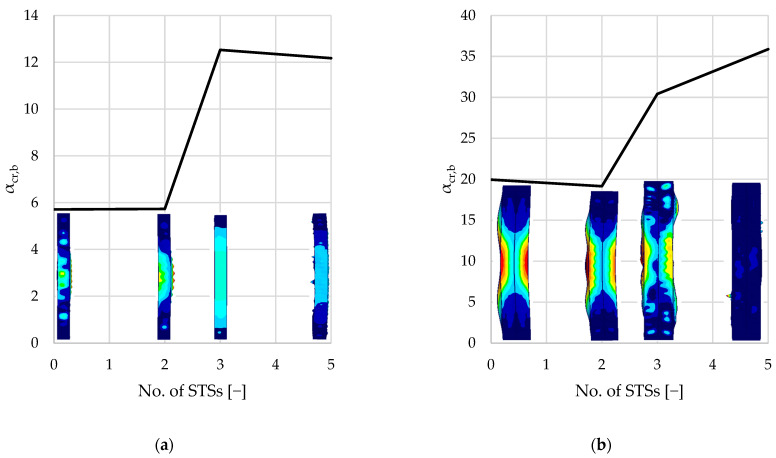
Diagrams and shapes of flexural buckling modes: (**a**) single-branched columns; (**b**) double-branched columns.

**Figure 12:**
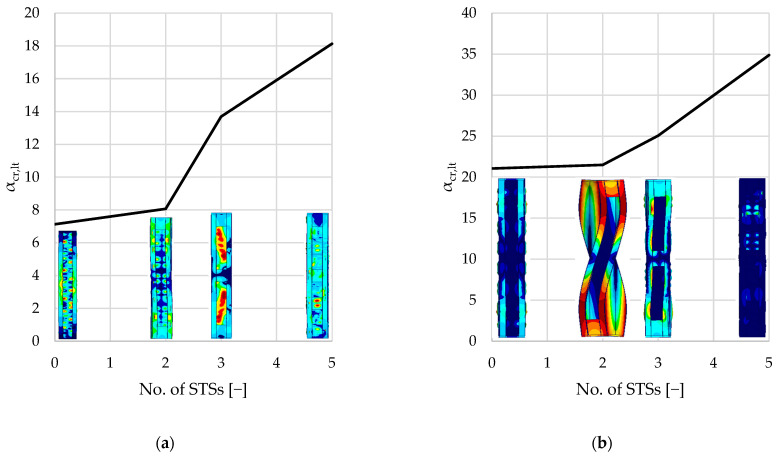
Diagrams and shapes of torsional buckling modes: (**a**) single-branched columns; (**b**) double-branched columns.

Percentage values presented in parentheses present the difference between the reference values resulting from unreinforced elements (samples A and A2). The results summarized in Table 3 are presented graphically in Figure 8, Figure 9, Figure 10, Figure 11 and Figure 12.

**Figure 13:**
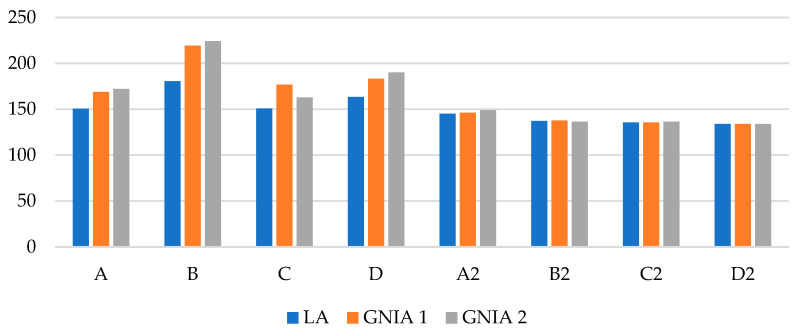
Column chart of von Mises stresses in columns resulting from LA and GNIA.

Table 4 presents the von Mises stresses measured in the midplane of the finite element. The values were read excluding the 4 cm zones near supports to avoid errors due to irrational stress concentrations. Stress values of the linear analysis (LA), geometrically nonlinear analysis considering local and flexural global imperfections (denoted as GNIA 1), and GNIA 2, which considers distortional and global flexural imperfections, are provided. Percentage differences noted in parentheses relate to results obtained from the linear analysis. The results seen in Table 4 are graphically presented in Figure 13.

## 4. Discussion

As seen in Figure 8 and Table 3, local buckling is associated with the lowest critical load factor (1.3–1.5 and 6–7, respectively, for single- and double-branched columns) for the first buckling mode. After local buckling, distortional buckling emerges as the next significant mode at $\alpha_{cr}$, ranging from 3.8 to 7.4 for single-branched and 13.7 to 21.8 for double-branched columns. Flexural buckling is associated with $\alpha_{cr}$, ranging from 5.7 to 12.5 and 19.1 to 35.9, respectively, for single- and double-branched columns. Torsional buckling mode is associated with $\alpha_{cr}$, ranging from 7.1 to 18.1 and 21.1 to 34.9, respectively, for single- and double-branched columns. As mentioned above, different kinds of loss of stability can be associated with different values of critical load. Oftentimes, there is a danger of interaction of different stability losses, e.g., distortional–flexural, local–distortional, etc.

Reinforcing the columns with sectional transverse stiffeners has a negligible influence on the local buckling capacity. In all cases, the local buckling occurred in the web of the column, which was not reinforced in any way. For double-branched columns, the critical load factor related to local buckling was, on average, 4.17 times higher in comparison to single-branched columns. This difference is caused by the doubled thickness of webs in double-branched columns. Note that, in the model, webs of each C-section in double-branched columns are treated as one shell element. As seen in Figure 8a, for the single-branched columns, local buckling of the web appears along the entire length of the element with varying magnitude. For double-branched columns, presented in Figure 10b, local buckling of the web appears mainly in the region of the supports. On both single- and double-branched columns, buckling of the flanges with a smaller magnitude can be observed.

In terms of distortional buckling of the columns, adding two STSs on the ends has a negative influence on critical load factor $\alpha_{cr,dist}$. The reason for this behavior may be the folding of the edge lip parallel to the first fold. This reduces the inertia where the distortional mode has maximum amplitude. In order to increase the distortional buckling capacity of the columns, STSs in the span of the column are required. Results show that columns reinforced by three STSs (two at the ends and one in the middle of the column) increase the critical load factor related to distortional buckling modes. Further, for the analyzed columns, further reinforcement results in capacity gain of 15% for single-branched and 1% for double-branched columns.

Flexural buckling modes resulting from the analysis are an interaction of distortional buckling and flexural buckling; therefore, similarly to the distortional mode analysis, adding two STSs at the ends of the columns resulted in an insignificant change in the capacity compared to the unreinforced element. For single-branched columns, the capacity gain was smaller than 1%, and for the double-branched columns, a 4% capacity loss was observed. Adding the third STS in the middle of the column prevented the susceptibility of the element to distortional buckling, which has a considerable influence on the flexural buckling capacity. For the single-branched column, the critical load factor for three STSs is 218%. For the double-branched column, the difference is 60%.

For the examined axially loaded columns, torsional buckling modes are associated with the highest critical load factors compared to other loss-of-stability modes. Introducing STSs further increases the critical load factor. As noticed previously, two STSs provide no significant difference in capacity. Adding the third STS in the middle of the span resulted in a 70% increase in the critical load factor for the single-branched elements. Naturally, monosymmetric cross-sections are more susceptible to torsion than the bisymmetric and axisymmetric cross-sections. Reinforcing single-branched columns results in turning the cross-section into a bisymmetric one. For double-branched columns, applying STSs does not provide such a significant difference in torsional resistance.

Analysis requiring a subsequent design check—GNIA analysis in combination with LBA analysis—was performed, and the von Mises stresses in the midplane of the shell finite elements were compared to the stresses obtained in LA. For single-branched columns, the results from the GNIA analysis, considering a combination of local and global bow imperfections, were 12–21% higher than the results obtained with LA. In the case of considering distortional imperfection combined with global bow imperfection, the difference in results ranged from 8 to 24%. In the case of double-branched columns, the results obtained with GNIA analysis differed by less than 3% compared with the linear analysis. This suggests that double-branched columns are less susceptible to second-order effects. The number of STS elements influences the values of stresses and the differences in results from linear and nonlinear analyses. The random nature of imperfections and the unevenness of magnitudes observed in the different columns causes the results to not follow a pronounced trend.

## 5. Conclusions

This article examines single- and double-branched sections subjected to axial loading. Such elements can serve as components in support structures for industrial platform decks that carry equipment or HVAC units, or simply as working platforms. In previous work [23], beam elements of this type were investigated. In the future, the authors aim to develop frame systems composed of such elements.

This paper described important aspects of numerical design calculation of thin-walled columns, as well as a novel method of reinforcing the thin-walled cold-formed steel columns with sectional transverse strengtheners. The validity of the concept was determined through linear buckling analysis (LBA), as well as geometrically nonlinear analysis considering imperfections (GMNIA). Elastic load factors *α_cr_* and the von Mises stresses were compared. The results of the numerical calculations confirmed the effectiveness of the proposed strengthening method for cold-formed columns. The addition of sectional transverse stiffeners (STSs) has a mode-dependent influence on the load-bearing capacity of columns, with negligible effects on local buckling due to unreinforced webs. For distortional, flexural, and torsional buckling, two end STSs often yield insignificant or negative changes, but adding a mid-span STS (e.g., three total) significantly enhances the capacity—up to 15% for distortional, 218% for flexural, and 70% for torsional in single-branched columns—while gains are smaller (1–60%) in double-branched ones. STSs also reduce the susceptibility to second-order effects, particularly in single-branched columns, by promoting bisymmetric cross-sections, though imperfection variability limits clear trends.

The promising results of the proposed structural solution require further investigation. Future work related to current study will include precise connection modeling considering contact boundary conditions and PCM connections modeling. Complex numerical modeling may require more sophisticated software, e.g., ABAQUS CAE [27]. The thorough numerical analyses will require validation through an experimental analysis. Furthermore, the results of both experimental and numerical analyses will be verified with a comparison to analytical solutions.

## Figures and Tables

**Table 1:** List of cold-formed thin-walled column variants.

Type	Cross-Section	No. of STSs ^1^
A	single-branched	0
B		2
C		3
D		5
A2	double-branched	0
B2		2
C2		3
D2		5

^1^ STS—sectional transverse strengthening.

**Table 2:** Quantitative influence of mesh size on the analysis of a single-branch column.

Mesh Size [mm]	First Mode α_cr_ [−]	No. of Finite Elements [−]	Size of the Output File [MB]	Time of the Analysis [s]
100	246.119	340	1.15	12
80	231.751	476	1.56	17
60	210.35	612	2.01	22
50	202.359	680	2.25	22
40	190.405	898	2.95	29
35	187.379	1034	3.36	34
30	185.928	1182	3.85	39
25	185.007	1440	4.74	46
20	177.07	2300	7.56	75
15	175.845	3264	10.6	108
10	173.453	5800	19.2	192
5	172.329	20,000	66.2	862
3	172.122	54,108	179	2870

**Table 3:** Critical load factors related to each type of loss of stability.

Type	Cross-Section	No. of STSs ^1^	*α*_*cr*,*l*_		*α*_*cr*,*dist*_		*α*_*cr*,*b*_		*α*_*cr*,*t*_	
A	Single-branched	0	1.398		3.876		5.710		7.128	
B		2	1.495	(+7%)	4.567	(18%)	5.733	(0%)	8.069	(13%)
C		3	1.496	(+7%)	6.274	(+62%)	12.529	(+119%)	13.686	(+92%)
D		5	1.387	(−1%)	7.378	(+90%)	12.716	(+123%)	18.137	(+154%)
A2	Double-branched	0	7.143		13.708		19.945		21.058	
B2		2	6.376	(−11%)	13.389	(−2%)	19.147	(−4%)	21.486	(2%)
C2		3	6.340	(−11%)	21.682	(58%)	30.410	(+52%)	25.060	(+15%)
D2		5	6.089	(−15%)	21.869	(+60%)	35.890	(+80%)	34.890	(+66%)

^1^ STS—sectional transverse strengthening.

**Table 4:** Von Mises stresses in columns resulting from LA and GNIA.

Type of Element	LA [MPa]	GNIA [MPa]			
		1		2	
A	150.7	168.92	(+12%)	172.24	(+14%)
B	180.5	219.3	(+21%)	224.2	(+24%)
C	150.8	176.7	(+17%)	162.9	(+8%)
D	163.3	183.3	(+12%)	190.1	(+16%)
A2	145.1	146.3	(+1%)	149.0	(+3%)
B2	137.1	137.8	(+1%)	136.4	(−1%)
C2	135.6	135.5	(0%)	136.4	(+1%)
D2	133.9	133.8	(0%)	134.0	(+0%)

## Data Availability

The original contributions presented in this study are included in the article. Further inquiries can be directed to the corresponding authors.
